# Bioequivalence of a Generic Vortioxetine 20 mg Tablet in Healthy Mexican Volunteers Under Fasting Conditions

**DOI:** 10.3390/ph19071093

**Published:** 2026-07-16

**Authors:** Porfirio de la Cruz Cruz, Alberto Martínez Muñoz, Erika Gabriela Guido Ávila, Omar Emmanuel Hernández Piña, Abraham Escobedo-Moratilla, José Trinidad Pérez Urizar

**Affiliations:** 1 Doctorado Institucional en Ingeniería y Ciencia de Materiales (DICIM), de la Universidad Autónoma de San Luis Potosí (UASLP), Av. Sierra Leona No. 550 Col. Lomas 2da. Sección, San Luis Potosí C.P. 78210, Mexico; a365618@alumnos.uaslp.mx; 2 FS SCIENTIA PHARMA S.A. de C.V. (Authorized Third Party), Fray Diego de la Magdalena 630, Jardín, San Luis Potosí C.P. 78270, Mexico; alberto.martinez@fsscientiapharma.com; 3 Asofarma de México S.A. de C.V., Av. Santa Fe 485, Lomas de Santa Fe, Contadero, Cuajimalpa de Morelos, Ciudad de México C.P. 05348, Mexico; eguido@adium.com.mx (E.G.G.Á.); ophernandez@adium.com.mx (O.E.H.P.); aescobedo@adium.com.mx (A.E.-M.); 4 Facultad de Ciencias Químicas, de la Universidad Autónoma de San Luis Potosí, Martínez #6, Av. Dr. Manuel Nava, Zona Universitaria, San Luis Potosí C.P. 78210, Mexico

**Keywords:** generic drugs, bioequivalence, pharmacokinetics, in vitro dissolution, major depressive disorder

## Abstract

**Background/Objectives:** Major Depressive Disorder (MDD) is a complex psychiatric condition characterized by persistent low mood and cognitive dysfunction. Vortioxetine, a multimodal antidepressant, modulates serotonin receptor activity and inhibits the serotonin transporter. This study aimed to evaluate the bioequivalence and in vitro dissolution performance of a generic 20 mg vortioxetine formulation compared to the reference product under fasting conditions in healthy Mexican volunteers. **Methods:** This was a randomized, open-label, two-period, crossover study. Twenty healthy subjects were enrolled, and 18 completed both periods, with a 4-week washout period between them. Plasma concentrations were quantified using a validated LC-MS/MS method. **Results:** The 90% confidence intervals (CI) for the geometric mean ratios (Test/Reference) of C_max_ and AUC_0–72_ were 92.32% [84.51–100.85] and 96.77% [91.10–102.80], respectively. Both parameters fell within the 80.00–125.00% bioequivalence acceptance range. No serious adverse events were reported. **Conclusions:** The generic vortioxetine formulation is bioequivalent to the reference product, supporting its therapeutic interchangeability for the treatment of MDD. NCT07624357.

## 1. Introduction

Major Depressive Disorder (MDD) is one of the most common psychiatric disorders worldwide. It encompasses a range of emotional, cognitive, autonomic, and behavioral changes, including depressed mood, diminished interest and/or pleasure, difficulty concentrating and/or remembering, indecisiveness, feelings of guilt and hopelessness, suicidal thoughts, anxiety, pain or psychosomatic complaints, fatigue, psychomotor retardation, and changes in appetite and sleep patterns, resulting in a significant psychosocial impact [1].

Vortioxetine is a multimodal antidepressant approved for the treatment of MDD. It differs from traditional selective serotonin reuptake inhibitors (SSRIs) and serotonin-norepinephrine reuptake inhibitors (SNRIs) by combining serotonin reuptake inhibition with targeted modulation of various presynaptic and postsynaptic 5-HT receptors [2,3,4]. This complex mechanism is highly beneficial in treating both the emotional and cognitive symptoms of depression [5,6], while simultaneously offering a favorable tolerability profile [7].

Following the oral administration of a single 20 mg dose under fasting conditions, vortioxetine exhibits a relative bioavailability of 75% [8], with peak plasma concentrations (approximately 8.11 ng/mL) typically achieved within 7 to 11 h [9]. Its absorption is unaffected by concomitant food intake [9], and the drug is a weak substrate for the P-glycoprotein transporter [10]. Vortioxetine is widely distributed to all organs—including the liver and kidneys—crosses the blood-brain and placental barriers, and is detectable in breast milk [11,12]. It exhibits high protein binding (99%) and an apparent volume of distribution (Vd) of 2500–3400 L, indicating extensive extravascular distribution [10,13].

The drug is extensively metabolized by cytochrome P450 (CYP) enzymes, which catalyze its oxidation, and by uridine diphosphate glucuronosyltransferase (UGT), which mediates subsequent glucuronidation. The primary CYP450 enzyme involved in this biotransformation is CYP2D6, supported by CYP3A4/5, CYP2A6, CYP2C9, and CYP2C19. Six vortioxetine metabolites have been observed in plasma; however, Lu AA34443 is the main circulating metabolite, accounting for 14–28% of the plasma metabolite composition over 72 h after a single dose. This major metabolite is pharmacologically inactive, as it does not bind to the primary 5-HT receptors [14,15].

Vortioxetine is predominantly eliminated via hepatic metabolism (approximately 99% of total clearance), with renal clearance of the parent drug contributing less than 1%. Following oral administration of radiolabeled vortioxetine, the drug-related material is primarily excreted as inactive metabolites in urine (59%) and feces (26%), with only a negligible amount (1.5%) of the unchanged drug detected [9]. Urinary acidification does not affect the reabsorption of the drug or its metabolites. The elimination half-life is approximately 83.35 ± 56.39 h [16].

While the clinical efficacy of vortioxetine in MDD is well-established, the scientific rationale for rigorously evaluating new generic formulations in vivo is fundamentally linked to its biopharmaceutical properties. According to the FDA, vortioxetine is a Biopharmaceutics Classification System (BCS) Class III compound, characterized by high solubility but low permeability [17,18]. For BCS Class III drugs, absorption is primarily permeability-limited; therefore, formulation-related factors—such as excipient composition and manufacturing characteristics—may significantly influence drug absorption and systemic exposure. Consequently, although comparative in vitro dissolution profiles provide important information regarding product behavior, dissolution data alone may not fully predict in vivo performance, and insufficiently sensitive or overly discriminatory in vitro dissolution methods may lead to false-positive or false-negative bioequivalence conclusions [19,20]. In this context, in vivo bioequivalence studies remain the gold standard for directly demonstrating comparable systemic exposure between products. In accordance with the regulatory requirements of the Mexican health authority (COFEPRIS) and the national guideline NOM-177-SSA1-2013 [21], the demonstration of bioequivalence through a robust in vivo pharmacokinetic study is strictly mandated for oral drug products containing vortioxetine.

While bioequivalence studies are primarily designed to fulfill regulatory requirements for therapeutic interchangeability, they also provide valuable baseline pharmacokinetic data for specific demographic groups. Polymorphic enzymes, notably CYP2D6, extensively metabolize vortioxetine. Although the present regulatory study did not include pharmacogenetic profiling, establishing baseline pharmacokinetic parameters for vortioxetine in a healthy Mexican population offers critical empirical data for future investigations. Such studies are necessary to fully elucidate the impact of regional phenotypic variations on vortioxetine disposition.

## 2. Results

### 2.1. Demographic Characteristics

**Figure 1:**
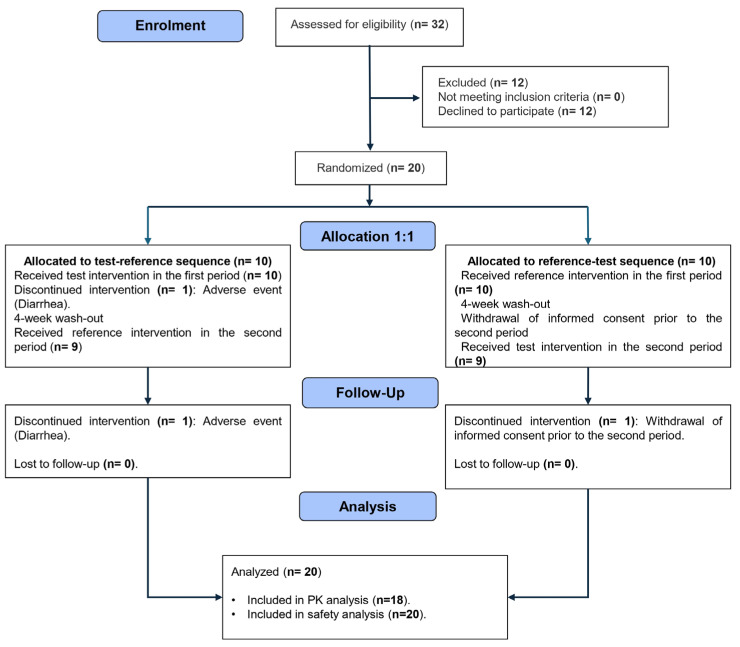
Study flowchart. n: number of subjects; PK: pharmacokinetic.

Twenty healthy volunteers enrolled in the study and received at least one dose of the study medication. The cohort (n = 20) consisted of 8 females (40%) and 12 males (60%), with a mean age of 47 ± 10 years and a mean Body Mass Index (BMI) of 23.3 ± 2.0 kg/m^2^. Baseline characteristics were well-balanced, and no clinically significant abnormalities were noted during screening (Table 1). Two subjects subsequently discontinued the study, leaving 18 participants in the Per-Protocol Population. One subject was withdrawn after presenting with diarrhea at or before twice the median tmax value during Period 1, while another voluntarily withdrew informed consent during Period 2 (Figure 1).

### 2.2. In Vitro Dissolution Profiles

**Figure 2:**
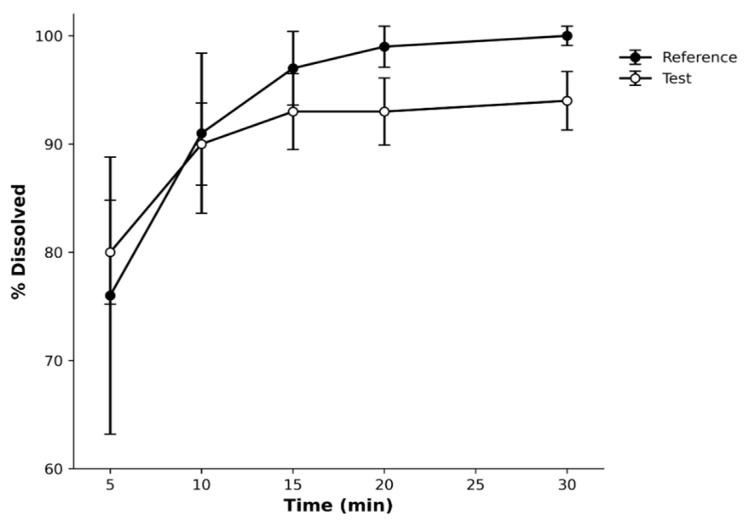
Mean comparative dissolution profiles of the 20 mg test formulation (batch 98283) and the reference formulation (Trintellix^®^, batch 12655210) in 900 mL of 0.1 HCl using USP Apparatus II at 50 rpm. Error bars represent the standard deviation (n = 12).

**Figure 3:**
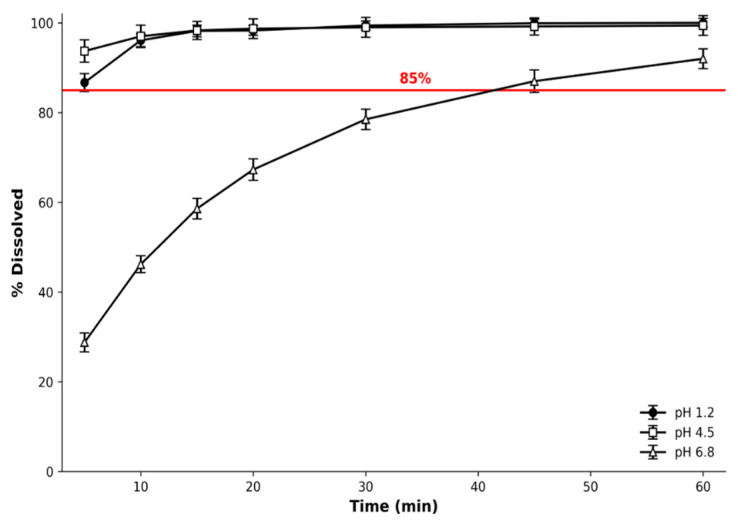
Mean dissolution profiles of the 20 mg test formulation (batch 98283) in 900 mL of dissolution media at pH 1.2, 4.5, and 6.8 using USP Apparatus II at 75 rpm. Error bars represent the standard deviation (n = 12).

The comparative dissolution profiles of the 20 mg test and reference formulations in 0.1 N HCl are presented in Figure 2. Both formulations exhibited very rapid and similar dissolution behavior, achieving more than 85% dissolved vortioxetine within 15 min. According to current regulatory criteria (e.g., ICH M9 and NOM-177-SSA1-2013) [18,22], dissolution profiles meeting this condition (≥85% in 15 min) are considered similar and do not require a formal mathematical comparison using the similarity factor (f2). These highly comparable in vitro findings were consistent with the observed in vivo bioequivalence between the formulations. The dissolution behavior of the 20 mg test formulation under biowaiver conditions is presented in Figure 3. Very rapid dissolution was observed in pH 1.2 and pH 4.5 media, with >85% of vortioxetine dissolved at 15 min under both conditions. In contrast, dissolution at pH 6.8 was considerably slower, reaching a mean dissolved fraction of 78.5% at 30 min and 87.0% at 45 min; thus, it did not meet the criteria for rapid or very rapid dissolution according to the current ICH M9 guideline. Nonetheless, when the 20 mg formulation was compared with the lower-strength formulations at pH 6.8, all dissolution profile comparisons met the similarity criteria (f2 ≥ 50), yielding f2 values of 71, 68, and 87 for the 5, 10, and 15 mg products, respectively. Notably, the same 20 mg batch was also shown to be bioequivalent in vivo, suggesting that in vitro dissolution behavior alone may not fully predict the performance of vortioxetine formulations.

### 2.3. Pharmacokinetics

**Figure 4:**
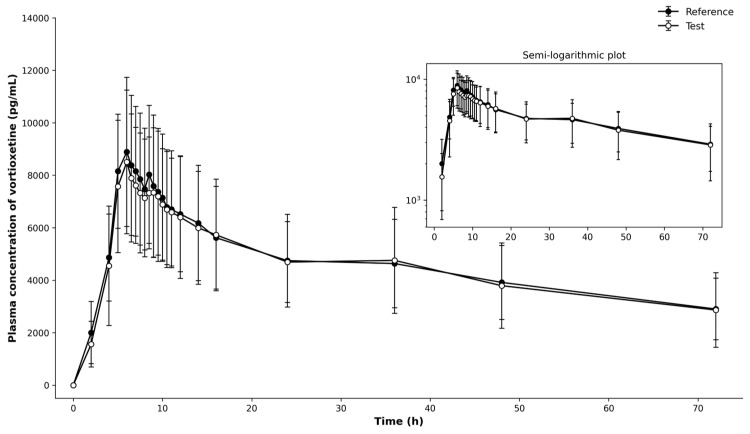
Mean ± SD plasma concentration-time profile of vortioxetine after a single oral dose of a 20 mg tablet. Reference (black circle), test (white circle).

The mean plasma concentration-time profiles for both formulations were nearly superimposable, as illustrated in Figure 4. Key pharmacokinetic parameters, C_max_, t_max_, and AUC_0–72_ are summarized for the test and reference products in Table 2. The pharmacokinetic (PK) parameters for the test and reference formulations are summarized (expressed as mean ± SD) as follows: 8668.86 ± 2848.31 pg/mL, 6.33 ± 1.30 h and 317,529.07 ± 119,053.25 pg·h/mL, and 9309.12 ± 2781.03 pg/mL, 6.06 ± 1.24 h and 324,396.84 ± 110,130.54 pg·h/mL, for C_max_, t_max_ and AUC_0–72_ of the test and reference formulation, respectively. Statistical analysis using ANOVA on log-transformed data revealed no significant effects of sequence, period, or treatment (*p* > 0.05) for the primary pharmacokinetic parameters evaluated for vortioxetine. Therefore, the statistical bioequivalence assessment conducted for vortioxetine 20 mg film-coated tablets was considered valid.

To ascertain the bioequivalence of the test and reference vortioxetine products, the geometric mean ratios and their corresponding 90% confidence intervals (CIs) were calculated for the log-transformed primary parameters. As detailed in Table 3, the resulting 90% CIs were 84.51–100.85% for C_max_ and 91.10–102.80% for AUC_0–72_. Because these values strictly fall within the conventional regulatory acceptance thresholds of 80.00% to 125.00%, the therapeutic equivalence of the formulations is statistically confirmed. Furthermore, the statistical power of the analysis, combined with the observed intra-subject variability (15.2% for C_max_ and 10.3% for AUC_0–72_), demonstrated that the chosen crossover design was highly sensitive and appropriate for assessing a molecule with an extended elimination half-life.

### 2.4. Safety and Tolerability Analysis

Both formulations were well-tolerated. A total of 14 adverse events (AEs) were reported by 9 subjects (45% of the total study population) during the trial (Table 4). All AEs were classified as mild or moderate in intensity and resolved without clinically significant complications. The most frequent AEs were headache and diarrhea, which are consistent with the known safety profile of vortioxetine. Of the total AEs, 6 (43%) were associated with the test formulation (experienced by 5 subjects), and 8 (57%) occurred with the reference drug (experienced by 6 subjects). Regarding gender distribution, 4 (28%) events occurred among male participants and 10 (72%) among female participants. In terms of causality, 13 (93%) events were classified as probably related to the study drug, and 1 (7%) as possibly related. No serious adverse events (SAEs) or dropouts due to toxicity occurred during the study.

## 3. Discussion

Vortioxetine is a multimodal antidepressant characterized by complex pharmacology and a Biopharmaceutics Classification System (BCS) Class III designation (high solubility, low permeability) [23]. Given these physicochemical properties, regulatory authorities strictly require that bioequivalence be established for generic alternatives to support therapeutic interchangeability and ensure comparable in vivo performance.

The results of this study successfully demonstrate that the 20 mg generic formulation is bioequivalent to the reference product (Trintellix^®^) under fasting conditions. Beyond fulfilling regulatory requirements, a critical objective of this investigation was to establish a robust pharmacokinetic baseline for vortioxetine in a Latin American population, which remains underrepresented in the scientific literature. When systematically comparing the pharmacokinetic profile obtained in our healthy Mexican cohort (mean C_max_ ranging from 8.67 to 9.31 ng/mL; t_max_ of ~6 h) with previously published data from diverse ethnic populations, a notable cross-regional consistency emerges. Chen et al. reported a comparable C_max_ of 8.11 ng/mL and a t_max_ of 7–11 h for a 20 mg oral dose [9], while Fagiolini et al. observed a C_max_ of 7.52 ng/mL and a median t_max_ of 6 h using the immediate-release tablet formulation [24]. Furthermore, our results align closely with systemic exposure metrics reported in Chinese [16] and Japanese [14] subjects evaluated under equivalent single-dose conditions. Collectively, these findings indicate that the absorption rate and systemic exposure of vortioxetine in healthy Mexican subjects fall within the range consistently reported across Caucasian and Asian populations.

Evidence from a comprehensive clinical pharmacokinetic evaluation by Chen et al. indicates that race/ethnicity does not have a clinically meaningful impact on vortioxetine pharmacokinetics [9]. This conclusion is further supported by the findings of Matsuno et al. [14] and Bai et al. [16], who independently demonstrated pharmacokinetic profiles in Japanese and Chinese subjects, respectively, that were comparable to those previously reported in Western populations. Therefore, rather than revealing clinically meaningful population-specific pharmacokinetic differences, the present study extends the available evidence to a previously underrepresented Latin American population, demonstrating that vortioxetine exhibits a consistent pharmacokinetic profile across geographically and ethnically distinct populations under fasting conditions. These findings expand the available pharmacokinetic evidence in Latin American populations and provide the first published pharmacokinetic data derived from a bioequivalence study of vortioxetine in healthy Mexican volunteers. This cross-population consistency is particularly scientifically relevant given that vortioxetine is extensively metabolized by CYP2D6 [14], an enzyme characterized by profound global phenotypic variability (e.g., varying prevalences of poor and ultrarapid metabolizers). The demonstration that the systemic exposure in the Mexican cohort strongly overlaps with European and Asian ranges suggests that underlying ethnic differences in the distribution of CYP2D6 polymorphisms do not result in clinically meaningful differences in overall single-dose exposure. Consequently, these empirical data further support the consistency of vortioxetine pharmacokinetics across diverse populations and contribute additional clinical evidence from a Latin American cohort.

From a mechanistic perspective, these findings also highlight that in vitro dissolution behavior alone may not fully capture the in vivo absorption kinetics of vortioxetine. Because the present study was designed exclusively to evaluate regulatory pharmacokinetic bioequivalence, the specific mechanistic drivers of vortioxetine’s absorption such as permeability-limited absorption or distribution-related processes, including lysosomal sequestration [25,26,27], cannot be definitively confirmed from the available clinical data. These theoretical pathways remain hypotheses for future investigation, where physiologically based biopharmaceutics modeling (PBBM) could serve as a valuable tool to explore their relative contributions to systemic exposure.

The achievement of bioequivalence in this study is particularly significant given the BCS Class III classification of vortioxetine [17,18]. For such compounds, the rate and extent of absorption may be influenced by permeability-related processes, making the evaluation of formulation equivalence highly relevant. The 90% confidence interval for both C_max_ (84.51–100.85%) and AUC_0–72h_ (91.10–102.80%) fell well within regulatory limits, confirming that the excipients used in the generic formulation did not result in clinically meaningful differences in the rate or extent of vortioxetine absorption compared with the innovator product. This observation is particularly relevant because the test formulation is not identical to the reference product, qualitatively and quantitatively (Q1/Q2). This finding aligns with previous reports indicating that although BCS Class III drugs are theoretically more sensitive to excipient effects, clinically meaningful alterations in drug absorption are generally associated with specific absorption-modifying excipients administered at sufficiently high concentrations, particularly osmotically active agents such as sorbitol or mannitol [28]. Moreover, conducting the study under fasting conditions provided a highly sensitive setting for detecting potential formulation-related differences while minimizing physiological variability.

The implementation of a truncated sampling schedule (AUC_0–72_) was scientifically justified by the drug’s long elimination half-life of approximately 83 h. For such long-half-life drugs, the absorption phase is the most sensitive period for detecting potential formulation-related differences. Therefore, in accordance with current FDA [29], EMA [30], and Mexican regulatory guidelines (NOM-177-SSA1-2013) [21], AUC_0–72_ serves as a robust and reliable surrogate for total exposure (AUC_0–∞_), avoiding unnecessary prolonged blood sampling while ensuring a rigorous bioequivalence assessment.

Additionally, while vortioxetine is utilized globally, pharmacokinetic literature specifically evaluating Latin American cohorts remains scarce. Vortioxetine is extensively metabolized by polymorphic enzymes, notably CYP2D6 [14]. Although the present study was undertaken for regulatory purposes and did not include pharmacogenetic profiling, establishing baseline pharmacokinetic parameters for vortioxetine in a healthy Mexican population provides valuable foundational data. This baseline is critical for future pharmacogenetic studies investigating the potential impact of CYP2D6 phenotypic variation on vortioxetine disposition in this demographic [31].

All adverse events reported during the study were categorized as mild or moderate in intensity. None of the events required pharmacological intervention, and all participants recovered fully without sequelae or complications. The safety profile observed in this trial is consistent with findings from previous bioequivalence studies evaluating single 20 mg doses of vortioxetine [16,23].

The clinical implications of these findings are significant. While the single-dose concentrations observed in this study are below the steady-state therapeutic range (10–40 ng/mL) [31,32], the established bioequivalence in the rate and extent of absorption supports the expectation of comparable systemic exposure during chronic administration. Given the long elimination half-life of vortioxetine (83 h) [16], the truncated single-dose design used in this study remains scientifically appropriate and sufficiently sensitive to detect potential formulation-related differences in systemic exposure. Consequently, patients switching from the innovator to the generic formulation would be expected to achieve comparable steady-state exposure and therapeutic responses [22]. From a public health perspective, validating the therapeutic interchangeability of this formulation facilitates broader access to multimodal antidepressants in emerging healthcare systems, ultimately supporting improved clinical management of MDD.

Study limitations: First, this study was conducted under fasting conditions in healthy volunteers; therefore, extrapolating these findings directly to patients with MDD relies entirely on the established regulatory framework for bioequivalence, rather than on direct comparative clinical efficacy outcomes. Second, the evaluation was limited to single-dose administration in strict accordance with regulatory guidelines. Finally, no pharmacogenetic characterization (e.g., CYP2D6 genotyping) was performed; thus, the specific impact of genetic polymorphism on the observed inter-subject variability remains a background hypothesis to be explored in future clinical research.

## 4. Materials and Methods

### 4.1. Drug Products

The test formulation was a coated tablet with an immediate-release (IR) mechanism containing 20 mg of vortioxetine (Lot No. 98283, expiry date July 2027), manufactured by Monte Verde S.A. (Pocito, San Juan Province, Argentina) and imported by Asofarma de México, S.A. de C.V. (Mexico City, Mexico). The reference formulation was Trintellix^®^, an IR tablet containing 20 mg of vortioxetine (Lot No. 12655210, expiry date April 2027), manufactured by Lundbeck (IL 60015) (Deerfield, IL, USA) for Takeda Pharmaceuticals USA Inc. (Lexington, MA, USA).

### 4.2. In Vitro Dissolution Studies

Comparative in vitro dissolution studies were conducted to support both the bioequivalence assessment of the 20 mg formulation and the biowaiver justification for the additional lower strengths (5, 10, and 15 mg), in accordance with Mexican regulatory requirements and internationally recognized principles for additional strength biowaivers described in FDA 21 CFR 320.22(d) [21,33]. Initially, the dissolution profiles of the 20 mg test formulation (batch 98283)—used in the in vivo bioequivalence study—and the reference formulation (Trintellix^®^, Lundbeck; batch 12655210) were compared. Subsequently, the same 20 mg test batch used in the clinical study served as the “reference” comparator for the dissolution profile assessment against the lower-strength formulations intended for biowaiver support. Additional comparative dissolution evaluations for the lower strengths were performed at pH 4.5 and pH 6.8.

Dissolution testing was performed using a USP Apparatus II Elite 8 dissolution tester (Hanson Research, Chatsworth, CA, USA). For the comparative dissolution evaluation of the 20 mg test and reference formulations, dissolution conditions were established in accordance with the FDA Dissolution Methods Database [34] for vortioxetine tablets, utilizing 900 mL of 0.1 N HCl at 37.0 ± 0.5 °C with a paddle rotation speed of 50 rpm. Samples (10 mL) were collected at 5, 10, 15, 20, and 30 min without medium replacement. Twelve dosage units were evaluated for each formulation.

For the biowaiver evaluations of the lower strengths (5, 10, and 15 mg), additional comparative dissolution studies were conducted in accordance with the Colombian Guideline on Bioavailability and Bioequivalence of Pharmaceutical Products [35]. These utilized a USP Apparatus II (Classic 6 dissolution tester, Hanson Research, Chatsworth, CA, USA) at 75 rpm in 900 mL of 0.1 N HCl (pH 1.2), pH 4.5 acetate buffer, and pH 6.8 phosphate buffer maintained at 37.0 ± 0.5 °C. Samples (10 mL) were collected at 5, 10, 15, 20, 30, 45, and 60 min without medium replacement. Twelve dosage units were evaluated by formulation. Following filtration through Nylon Titan3™ 0.45 µm membrane filters, the dissolved vortioxetine was quantified at 226 nm using a Prominence LC-20AD SIL-20AC HPLC system coupled to an SPD-10A UV detector (Shimadzu, Kyoto, Japan).

Dissolution profile analyses were performed in accordance with the criteria described in the Mexican regulatory guideline NOM-177-SSA1-2013 for interchangeability assessments and in ICH M9 [24]. Similarity between dissolution profiles was evaluated using the similarity factor (f2) [36].

### 4.3. Study Design

To evaluate the bioequivalence of two 20 mg vortioxetine tablet formulations, a prospective, longitudinal, randomized, open-label, two-way crossover study was conducted. Twenty healthy Mexican adults (18–55 years) participated under fasting conditions, with a minimum washout period of four weeks separating the two dosing phases to ensure complete drug clearance before the subsequent treatment. The trial was performed at the contract research organization (CRO) site Axis Clinicals Latina, S.A. de C.V., in Mexico City, Mexico. The protocol was reviewed and approved by an independent ethics committee and the Mexican regulatory authority (COFEPRIS), in accordance with the Declaration of Helsinki and ICH Good Clinical Practice (GCP) guidelines.

Following a 10 h overnight fast, subjects received a single 20 mg oral dose of either the test or reference formulation with 250 mL of water. Water was restricted from 1 h before dosing until 4 h post-dose, except for the fluid given with the medication. Participants were confined for 36 h for intensive monitoring and standardized dietary control (2500 kcal). The 4-week washout period between treatment periods substantially exceeded the minimum washout period of seven terminal half-lives recommended by the Mexican regulatory guideline NOM-177-SSA1-2013 [18], considering the drug’s known 83 h elimination half-life. All adverse events (AEs)—including their duration, severity, causality, and outcome—were documented by the Principal Investigator or clinical staff. Participants remained under close medical supervision throughout the study.

Due to the long elimination half-life of vortioxetine (approximately 83 h), the area under the curve (AUC_0–72_) was used. This approach strictly aligns with current FDA, EMA, and Mexican regulatory guidelines for bioequivalence studies of long-half-life drugs (AUC) [21,29,30], providing a robust and reliable measure of absorption extent without requiring prolonged confinement or sampling periods.

### 4.4. Study Population

All volunteers provided written informed consent before undergoing any study-related procedures. The study enrolled healthy male and female subjects aged 18 to 55 years with a BMI between 18 and 27 kg/m^2^. Health status was confirmed during the screening visit through medical history, physical examination, 12-lead electrocardiogram (ECG), and clinical laboratory testing, including a complete blood count (CBC), blood chemistry, pregnancy testing, urinalysis, and toxicology screening. On Day 0, participants underwent repeat pregnancy and breath alcohol tests. Female subjects of childbearing potential were required to use a highly effective method of contraception during the study period. Key exclusion criteria included pregnancy or lactation, a history or evidence of drug or alcohol abuse, significant gastrointestinal disorders, or the use of prescription or over-the-counter (OTC) medications within 14 days of the study’s initiation.

### 4.5. Sample Size

The statistical power analysis and sample size determination were conducted to satisfy the requirements of the NOM-177-SSA1-2013 guideline, which mandates the use of the pharmacokinetic parameter with the largest intra-subject coefficient of variation (CV_intra_) [21]. Based on unpublished data from a prior 2 × 2 pilot trial with 11 research subjects (Study No. C1B03716, Asofarma de México S.A. de C.V., Ciudad de México, Mexico), the confidence intervals for C_max_ and AUC_0–72_ were 74.87–101.24% and 82.69–101.19%, and the CV_intra_ values were 19.4% and 12.9%, respectively.

Computations were performed using R software version 4.1.2 (Rcmdr package, 2021), applying a two-one-sided *t*-test (TOST) framework appropriate for a standard 2 × 2 crossover design [37,38,39]. A significance level (α = 0.05), a target statistical power of at least 80%, and a theoretical test-to-reference geometric mean ratio of 0.95 were utilized for the calculation.

Under these specific constraints, a minimum of 18 evaluable participants was required to appropriately establish bioequivalence within the standard 80.00–125.00% acceptance margins. To proactively mitigate the impact of potential early withdrawals or protocol deviations during the clinical phase, the cohort was conservatively expanded by two additional volunteers, resulting in a final enrollment target of 20 subjects.

### 4.6. Pharmacokinetic Assessment and Analytical Method

Serial blood samples (6 mL) were collected pre-dose (0 h) and at 2, 4, 5, 6, 6.5, 7, 7.5, 8, 8.5, 9, 9.5, 10, 10.5, 11, 12, 14, 16, 24, 36, 48, and 72 h post-administration. Because vortioxetine exhibits a long terminal half-life and formulation-related differences are expected to be most readily detected during absorption, a truncated sampling schedule extending up to 72 h was employed. This aligns with FDA, EMA, and COFEPRIS guidelines, which utilize AUC_0–72_ as a reliable surrogate for total exposure (AUC_0–∞_) for long-half-life drugs. Samples were drawn into K2EDTA tubes and centrifuged at 3500 rpm for 10 min at 4 °C. The separated plasma was stored at −70 ± 15 °C until analysis.

The bioanalytical method was fully validated in accordance with current international regulatory guidelines. The calibration curve demonstrated excellent linearity over the concentration range of 50.0 to 29,998.8 pg/mL for vortioxetine. Quality control (QC) samples at concentrations of 149.7, 6343.5, and 15,441.8 pg/mL were quantified using a validated high-performance liquid chromatography–tandem mass spectrometry (LC-MS/MS) method developed in accordance with Good Laboratory Practice (GLP) principles and Mexican regulation [21]. Intra-assay and inter-assay precision (expressed as CV%) were consistently below 15% (and below 20% at the LLOQ). Accuracy (expressed as absolute deviation) was consistently ≤10.8% across all quality control levels. No significant matrix effects were observed, and the analyte remained stable under all tested conditions. Supporting bioanalytical validation data—including assessments of selectivity, precision, accuracy, recovery, matrix effect, linearity, and stability—are provided in the Supplementary Materials.

Sample extraction was performed via solid-phase extraction (SPE), utilizing vortioxetine-d8 as the internal standard (IS). In brief, 50 µL of the IS solution was added to plasma aliquots, followed by 400 µL of a pretreatment solution. After vortex mixing for 10 s, samples were loaded onto Strata-X SPE (33 µm) cartridges that had been previously activated with 1 mL of methanol (100%) and equilibrated with 1 mL of deionized water. The cartridges were subsequently washed with deionized water, followed by a 5% methanol solution, and dried for 3 min before elution. Vortioxetine was then eluted using 400 µL of mobile phase, vortex-mixed for 10 s, and transferred into autosampler vials (20 µL) for chromatographic analysis.

Chromatographic separation was achieved under reversed-phase conditions using a Prominence LC-20AD SIL-20AC HPLC system (SHIMADZU, Kyoto, Japan) equipped with a C18 4 × 3 mm precolumn (PHENOMENEX, Torrance, CA, USA) and an ACE 3 C18 35 × 4.6 mm, 3 µm column (AVANTOR, Aberdeen, Scotland, UK) maintained at 35 °C. The mobile phase consisted of acetonitrile, methanol, and 0.1% formic acid (40:30:30 *v*/*v*) with a flow rate of 0.75 mL/min. The autosampler temperature was maintained at 5 °C.

Detection was performed using an API 4000 triple quadrupole mass spectrometer (SCIEX, Concord, ON, Canada) operated in multiple reaction monitoring (MRM) mode utilizing positive ionization (ESI+) with a TurboIonSpray source. The monitored transitions were *m/z* 299.2 → 150.0 for vortioxetine and *m/z* 307.2 → 153.2 for the IS. Data acquisition and chromatographic integration were performed using Analyst^®^ software version 1.6.2. Plasma concentrations of vortioxetine were determined from calibration curves constructed using the peak-area ratio of vortioxetine to the IS. Quantification was based on linear regression analysis utilizing a 1/x^2^ weighting factor.

### 4.7. Tolerability and Safety Assessments

Safety and tolerability were monitored continuously throughout the clinical phase. Adverse events (AEs) were captured via spontaneous reporting by the subjects and through direct open-ended questioning by the clinical staff at periodic intervals. All documented AEs were coded using the Medical Dictionary for Regulatory Activities (MedDRA) and were systematically classified by severity (mild, moderate, or severe) and causal relationship to the study medication.

Safety monitoring also included comprehensive physical examinations conducted at screening and before final discharge. Furthermore, vital sign measurements (including blood pressure, heart rate, and temperature) were strictly recorded pre-dose and at 9, 11, 22, 36, 48, and 72 h post-administration to ensure the continuous well-being of the participants.

### 4.8. Statistical Analysis

Demographic characteristics and the baseline comparability of the enrolled subjects were evaluated using standard descriptive statistics. Safety evaluations were conducted in the intention-to-treat (ITT) population (n = 20), whereas pharmacokinetic (PK) and bioequivalence (BE) assessments were restricted to the per-protocol population (n = 18), which comprised subjects who completed both clinical periods and provided evaluable data.

The primary PK endpoints designated for the bioequivalence determination were C_max_ and AUC_0–72_, with the time to maximum concentration (t_max_) recorded as a secondary metric. Recognizing the extended elimination half-life of vortioxetine (approximately 83 h), a 72 h truncated sampling strategy was prospectively adopted. Consequently, the terminal elimination rate constant (Kel), the area under the curve extrapolated to infinity (AUC_0–∞_), and t1/2 were deliberately excluded from the bioequivalence evaluation, as specified in the Mexican bioequivalence guideline NOM-177-SSA1-2013 [21].

All PK metrics were derived through non-compartmental analysis (NCA) employing Phoenix WinNonlin version 8.4 (Certara L.P., Radnor, NJ, USA). Peak exposure values (C_max_ and t_max_) were obtained directly from the observed plasma concentration-time profiles. The area under the curve (AUC_0–72_) was calculated using the linear trapezoidal method.

For the formal statistical demonstration of bioequivalence, the Schuirmann’s two-one-sided *t*-tests (TOST) procedure was applied to the log-transformed primary parameters, C_max_ and AUC_0–72_. Furthermore, an Analysis of Variance (ANOVA) was executed to evaluate potential sources of variation. The ANOVA model included sequence, period, and formulation (treatment) as fixed effects, and subject nested within sequence as a random effect. Following the stipulations of the Mexican regulatory guideline NOM-177-SSA1-2013 [21], 90% confidence intervals (CIs) for the test-to-reference geometric mean ratios (test/reference) of C_max_ and AUC_0–72_ were calculated. Therapeutic equivalence was concluded if the 90% CIs were contained within the predefined acceptance criteria of 80.00% to 125.00%.

## 5. Conclusions

This study demonstrated that the 20 mg immediate-release test formulation of vortioxetine is bioequivalent to the reference product under fasting conditions. Both formulations exhibited comparable rates and extents of absorption, alongside similar tolerability profiles, confirming highly efficient in vivo biopharmaceutical performance. By generating robust pharmacokinetic evidence within a Latin American cohort, these findings support the therapeutic interchangeability of the generic formulation. Consequently, it represents a reliable and accessible alternative for the clinical management of major depressive disorders.

## Figures and Tables

**Table 1:** Demographic baseline characteristics of study subjects.

Variable	General Population n = 20	Female n = 8	Male n = 12
(%)		40.00	60.00
Age (years) mean ± SD	47 ± 10	43.4 ± 12.7	49.3 ± 7.5
Weight (kg) mean ± SD	61.6 ± 8.8	56.8 ± 7.7	64.8 ± 8.2
Height (m) mean ± SD	1.63 ± 0.09	1.54 ± 0.06	1.68 ± 0.06
BMI (kg/m^2^) mean ± SD	23.3 ± 2.0	23.9 ± 1.7	22.9 ± 2.1

%: percentage; BMI: Body Mass Index; n: number of subjects; SD: standard deviation.

**Table 2:** Pharmacokinetic parameters of vortioxetine after test and reference dosing. Data are given as mean ± SD.

Parameter	Vortioxetine	
	Test	Reference
C_max_ (pg/mL)	8668.86 ± 2848.31	9309.12 ± 2781.03
t_max_ (h)	6.33 ± 1.30	6.06 ± 1.24
AUC_0–72_ (pg·h/mL)	317,529.07 ± 119,053.25	324,396.84 ± 110,130.54

AUC_0−72_: area under the plasma concentration curve from 0 to 72 h; C_max_: maximum plasma concentration; t_max_: time to reach C_max_.

**Table 3:** Geometric mean ratios and 90% confidence intervals for the pharmacokinetic parameters of vortioxetine.

Parameter	Geometric Mean Ratio (%)	90% CI		Schuirmann’s TOST		Power
		LL	UL	*p* < 80%	*p* > 125%	
**C_max_**	92.32	84.51	100.85	0.010	0.000	0.860
**AUC_0–72_**	96.77	91.10	102.80	0.000	0.000	1.000

%: percentage; AUC_0−72_: area under the plasma concentration curve from 0 to 72 h; CI: confidence interval; C_max_: maximum plasma concentration; LL: lower limit; UL: upper limit.

**Table 4:** Summary of adverse events.

Variable	Test	Reference	Overall
Number of AEs/n			
Overall	6	8	14
Study-Drug-related	6	8	14
Type of AEs			
Headache	2	3	5
Dyspepsia	1	1	2
Diarrhea	3	2	5
Skin rash	0	1	1
Abdominal pain	0	1	1
TOTAL	6	8	14

AEs, adverse events; n, number of AEs.

## Data Availability

The original contributions presented in this study are included in the article/Supplementary Material. Further inquiries can be directed to the corresponding author.

## Supplementary Materials

The following supporting information can be downloaded at: https://www.mdpi.com/article/10.3390/ph19071093/s1, Figure S1: Representative calibration curve obtained using analyte-to-internal standard peak area ratios, showing the linear relationship between concentration and response for vortioxetine in human plasma. Table S1: Measured concentrations and variability across three independent calibration runs for the evaluation of method linearity of vortioxetine in human plasma. Table S2: Calibration curve parameters for vortioxetine. Table S3: Measured concentrations and variability across three independent calibration runs for the evaluation of method linearity (50.0–20,240.4 pg/mL) of vortioxetine in human plasma. Table S4: Summary of bioanalytical method validation results for vortioxetine.

## Abbreviations

The following abbreviations are used in this manuscript:

5-HT	Serotonin
AEs	Adverse events
ANOVA	Analysis of variance
AUC	Area under the curve
BCS	Biopharmaceutical Classification System
CBC	Complete blood count
BMI	Body mass index
CI	Confidence interval
C_max_	Peak plasma concentration
COFEPRIS	Federal Commission for the Protection against Sanitary Risk
CRO	Contract Research Organization
CVintra	Intra-subject coefficient of variation
CYP	Cytochrome P450
ECG	Electrocardiogram
ESI+	Positive electrospray ionization
EMA	European Medicines Agency
FDA	Food and Drug Administration
GCP	Good Clinical Practice
ICH	International Council for Harmonization
IR	Immediate release
IS	Internal standard
ITT	Intention-to-treat
K_el_	Terminal elimination rate constant
LC-MS/MS	Liquid chromatography–tandem mass spectrometry
LL	Lower limit
MedDRA	Medical Dictionary for Regulatory Activities
MDD	Major Depressive Disorder
MRM	Multiple reaction monitoring
NCA	Non-compartmental analysis
OTC	Over the counter
PK	Pharmacokinetics
PP	Per-protocol
SAEs	Serious adverse events
SD	Standard deviation
SSRI	Selective serotonin reuptake inhibitors
SNRI	Serotonin-norepinephrine reuptake inhibitors
t_1/2_	Elimination half-life
TOST	Two one-sided *t*-tests
t_max_	Time to reach peak plasma concentration
UGT	Uridine diphosphate glucuronosyltransferase
UL	Upper limit
Vd	Apparent volume of distribution
