# Supplementary material for: Bioequivalence of a Generic Vortioxetine 20 mg Tablet in Healthy Mexican Volunteers Under Fasting Conditions
Source: Pharmaceuticals (Basel). 2026 Jul 16;19(7):1093. doi: 10.3390/ph19071093 (PMC13415081; doi:10.3390/ph19071093)
Supplement: Supplementary file 1 [file pharmaceuticals-19-01093-s001.zip › pharmaceuticals-4399184-supplementary.pdf]

1. Supplementary material: Bioanalytical Method Validation

Validation of an Analytical Method for the Determination of Vortioxetine in Human K_2EDTA Plasma by LC–MS/MS (Information extracted and summarized from the bioanalytical validation report)

1.1 Objective and regulatory framework

The aim of this study was the full validation of an analytical method for the determination of vortioxetine in human K_2EDTA plasma.

The bioanalytical method validation was conducted in accordance with the applicable regulatory requirements for bioequivalence studies in Mexico (NOM-177-SSA1-2013). The validation approach is consistent with internationally accepted principles described in regulatory guidelines, including those from the European Medicines Agency (EMA) and the U.S. Food and Drug Administration (FDA), as well as Good Laboratory Practice (GLP) standards.

1.2 Summary of the analytical method

Vortioxetine concentrations in human K_2EDTA plasma were determined using a validated LC-MS/MS method with vortioxetine-d8 as the internal standard (IS). Sample preparation was performed by solid-phase extraction (SPE). Quantification was based on analyte-to-internal standard peak area ratios, and calibration curves were constructed using least-squares linear regression with a $1/x^2$ weighting factor.

1.2.1 Linearity and calibration curve

Calibration standards were prepared in human plasma over the concentration range of 50.00-29998.8 pg/mL. Calibration curves were constructed using analyte-to-internal standard peak area ratios across the studied concentration range.

The calibration model showed excellent linearity, with a mean correlation coefficient (r) of 0.99978.

Table S1. Measured concentrations and variability across three independent calibration runs for the evaluation of method linearity of vortioxetine in human plasma.

Run	**Nominal concentration** (pg/mL)							
	**50.00**	**100.0**	**499.8**	**3509.9**	**7019.7**	**14039.4**	**23399.0**	**29998.8**
1	49.9	100.5	491.0	3667.9	6860.9	14149.9	23541.5	29317.1
2	51.5	93.7	506.3	3687.6	6984.2	13812.6	23649.6	29414.2
3	49.0	104.6	487.2	3556.5	6920.8	14448.0	22791.8	29924.3
**Mean**	50.13	99.60	494.83	3637.33	6921.97	14136.83	23327.63	29551.87

**SD**	1.266	5.505	10.111	70.693	61.658	317.901	467.182	326.170
**%CV**	2.5	5.5	2.0	1.9	0.9	2.2	2.0	1.1

SD: standard deviation; %CV: coefficient of variation (%).

Table S2. Calibration curve parameters for vortioxetine.

Run	Slope (m)	Intercept (b)	Correlation coefficient (r)
1	0.9873	69.7090	0.9998
2	0.9889	57.1574	0.9998
3	0.9915	41.9312	0.9997
**Mean**	0.98923	56.26585	0.99978

Figure S1. Representative calibration curve obtained using analyte-to-internal standard peak area ratios, showing the linear relationship between concentration and response for vortioxetine in human plasma.

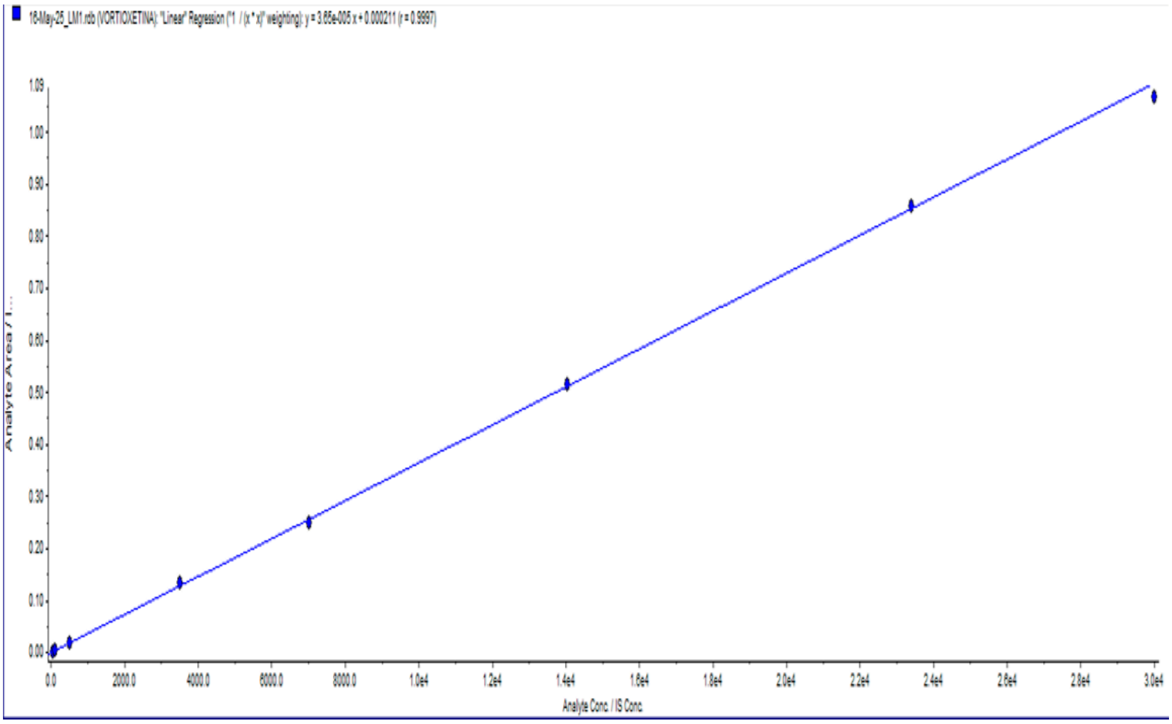

Linearity and reproducibility validation is also performed over a narrower range: 50.0–20,240.4 pg/mL.

The calibration model showed excellent linearity, with a mean correlation coefficient (r) of 0.99937.

Table S3. Measured concentrations and variability across three independent calibration runs for the evaluation of method linearity (50.0-20240.4 pg/mL) of vortioxetine in human plasma.

**Run**	**Nominal concentration** (pg/mL)							
	**50.00**	**100.0**	**500.1**	**2170.8**	**4341.6**	**8683.1**	**15787.5**	**20240.4**
1	51.0	96.9	476.2	2230.4	4469.4	8554.3	16403.7	19804.4
2	50.6	98.3	481.2	2176.4	4498.3	8550.5	16537.0	19678.7
3	51.3	95.8	471.7	2293.3	4430.7	8550.1	15800.1	20461.1
**Mean**	50.97	97.00	476.37	2233.57	4466.13	8551.63	16246.93	19981.40
**SD**	0.351	1.253	4.752	58.814	33.918	2.318	392.667	420.160
**%CV**	0.69	1.29	1.00	2.63	0.76	0.03	2.42	2.10

SD: standard deviation; %CV: coefficient of variation (%).

1.2.2 Accuracy and precision

Intra-day and inter-day accuracy and precision were evaluated using quality control (QC) samples at multiple concentration levels.

For repeatability, six replicates of the lower limit of quantification (50.1 pg/mL), as well as plasma quality control (QC) samples at low, medium, and high concentrations (149.7, 9660.6, and 22811.3 pg/mL), were analyzed in sextuplicate. In addition, six replicates of the highest calibration standard diluted at ratios of 1/2 and 1/4 were evaluated.

For reproducibility, six replicates of the lower limit of quantification (50.1 pg/mL) and plasma quality control samples at low, medium, and high concentrations (149.7, 9660.6, and 22811.3 pg/mL) were analyzed in sextuplicate, with three determinations performed by each analyst on at least two different days.

The method reproducibility is also validated over a narrower range (50.0–20,240.4 pg/mL): For reproducibility, six replicates of the lower limit of quantification (50.1 pg/mL) and plasma quality control samples at low, medium, and high concentrations (149.8, 6346.2, and 15,448.4 pg/mL) were analyzed in sextuplicate.

The method showed adequate precision, with coefficients of variation (CV) below 15%, and accuracy expressed as absolute deviation (AD) within 15% of nominal concentrations across all QC levels. For the lower limit of quantification (LLOQ), both precision (CV ≤ 20%) and accuracy (AD ≤ 20%) were within the predefined acceptance criteria.

1.2.3 Selectivity and matrix effect

Selectivity was evaluated using multiple individual plasma sources, including hemolyzed and lipemic samples, as well as plasma containing commonly co-administered drugs.

No significant interference was observed at the retention times of vortioxetine and the internal standard (IS). Matrix effects were assessed and demonstrated acceptable variability, with normalized matrix factor CV values below 15% at both QC (quality control) levels.

1.2.4 Recovery

Extraction recovery was evaluated at different concentration levels. The method showed consistent recovery across the concentration range for both vortioxetine and the internal standard, with CV values below 15%. The mean relative recovery of Vortioxetine was 80.62%.

1.2.5 Lower limit of quantification (LLOQ)

The LLOQ was established at 50.00 ng/mL, demonstrating acceptable accuracy and precision (CV ≤ 20% and absolute deviation ≤20%), confirming the suitability of the method for quantifying low plasma concentrations.

1.2.6 Stability

Stability of vortioxetine in plasma was evaluated at two concentration levels in human plasma analyzed in sextuplicate (149.7 and 22811.3 pg/mL), under various conditions, including:

- Short-term (room temperature) stability (23.42 hours)
- Autosampler stability (5°C, 51.08 hours).
- Freeze–thaw cycles (6 cycles, -70°C).
- Long-term storage stability (-70°C, 72 days).
- Refrigerated processed sample stability (47.93 hours)
- Working solution stability (short- and long-term) (23.00 h and 7 days)
- Stock solution stability (short- and long-term) (22.92 h and 7 days)
- Whole blood stability. (3.92 hours)

Virtioxetien was found to be stable under all tested conditions, with precision (CV ≤ 15% and accuracy expressed as absolute deviation ≤ 15%) within the predefined acceptance criteria.

1.3 Overall validation performance

The validation results demonstrated that the analytical method met all predefined acceptance criteria for bioanalytical method validation. The method showed adequate linearity, precision, accuracy expressed as absolute deviation, selectivity, recovery, sensitivity, and stability under all evaluated conditions.

Table S4. Summary of bioanalytical method validation results for vortioxetine.

Parameter	Acceptance criteria	Result
Linearity	r ≥ 0.98	0.99978
Precision (intra-day)	CV ≤ 15%	0.5-0.7%
Accuracy (intra-day)	Absolute deviation (AD) ≤15%	2.4- 5.9%
Precision (inter-day)	CV ≤ 15%	0.9-3.8%
Accuracy (inter-day)	AD ≤15%	1.3-10.8%
Selectivity	No interference at the retention time of the analyte and internal standard	No interference was observed at the retention time of vortioxetine and the IS in blank, hemolyzed, and lipemic plasma, or in the presence of tested drugs (salicylic acid acetaminophen, ibuprofen, ketorolac, ondansetron, loratadine, metformin, metamizole, loperamide)
LLOQ (50.1 pg/mL)	CV ≤ 20%, AD ≤20%	CV 7.7%, AD 7.1
Carryover	Carryover at the analyte retention time < 20% and carryover at the internal standard (IS) retention time < 5%	1.1% for vortioxetine and 0.0% for vortioxetine-d8
Recovery	CV ≤ 15%	1.3% (vortioxetine), 1.5 % (vortioxetine-d8)

Reinjection reproducibility	CV ≤ 15%, AD ≤ 15%	Stable up to 23.23 h (CV 0.4-1.8%, AD 1.6–2.6%)
Matrix effect	Normalized matrix factor CV ≤ 15% at both QC levels	1.4% (low QC), 2.2% (high QC)
Stability (short-term, room temperature)	CV ≤ 15%, AD ≤ 15%	Stable up to 23.42 h (CV 4.2%-4.9%, AD 6.0%-7.4%)
Stability (autosampler at 5 °C)	CV ≤ 15%, AD ≤ 15%	Stable up to 51.08 h (CV 0.4%-2.0%, AD 0.0%-2.1%)
Freeze–thaw stability (-70°C, 6 cycles)	CV ≤ 15%, AD ≤ 15%	Stable for 6 cycles (CV 0.2%-3.2%, AD 0.2%-3.6%)
Long-term stability (-70°C, 72 days)	CV ≤ 15%, AD ≤ 15%	
Refrigerated processed sample stability (2-8°C)	CV ≤ 15%, AD ≤ 15%	Stable up to 47.93 h (CV 0.6-0.8%, AD 0.9-2.1%)
Working solution stability (short- and long-term)	AD ≤ 10%	Stable up to 23.00 h (high, short-term), 23.07 h (low, short-term), and 7 days (high and low,long-term)<br>CV 0.8–0.9% and AD 0.6-1.3% (high, short-term); CV 0.6-1.2% and AD 1.8-2.8% (low, short-term); CV 0.8–1.0% and AD 0.3–0.7% (high, long-term); CV 0.8–0.8% and AD 2.7-2.9% (low, long-term)
Stock solution stability (short- and long-term)	AD ≤ 10%	Stable up to 22.92 h (short-term) and 7 days (long-

		term); CV 0.5–0.6% and AD 1.4–1.5% (short-term); CV 0.7–0.8% and AD 4.2–4.8% (long-term)
Whole blood stability	CV ≤ 15%, AD ≤ 15%	Stable up to 3.92 h (CV 0.5–2.5%, AD 4.8–6.0%)
